# Firsekibart for steroid withdrawal in glucocorticoid-dependent refractory gout: a case report

**DOI:** 10.3389/fimmu.2026.1785692

**Published:** 2026-03-24

**Authors:** Jinhui Tan, Hai Huang, Linghua Tan, Bo Li, Ruonan She, Shunji Long

**Affiliations:** 1 Department of Rheumatology and Immunology, People’s Hospital of Longhua, Shenzhen, Guangdong, China; 2 Department of Health Management, People’s Hospital of Longhua, Shenzhen, Guangdong, China; 3 Department of Health Management, Jiangmen Wuyi Hospital of Chinese Medicine, Jiangmen, Guangdong, China; 4 Department of Gynaecology, People’s Hospital of Longhua, Shenzhen, Guangdong, China

**Keywords:** antibodies, monoclonal, drug therapy, glucocorticoids, gout, interleukin-1beta

## Abstract

Glucocorticoid dependence presents a major therapeutic hurdle in refractory gout, often preventing successful steroid tapering and perpetuating a cycle of recurrent flares. We describe a 53-year-old male with severe tophaceous gout and glucocorticoid dependence, experiencing frequent flares upon reducing methylprednisolone below 16 mg/day despite urate-lowering therapy (ULT). After conventional anti-inflammatory agents proved inadequate, a single 200-mg subcutaneous dose of firsekibart (formerly known as genakumab), an anti-interleukin-1β (IL-1β) monoclonal antibody, was administered. Following this intervention, gout flares ceased immediately, enabling a glucocorticoid taper and eventual discontinuation over the following two months. Inflammatory markers normalized, renal function stabilized, and serum urate decreased. This case demonstrates the potential of firsekibart as a potent steroid-sparing agent, effectively breaking the cycle of glucocorticoid dependency in refractory gout and enabling successful long-term management.

## Introduction

Gout is the most common inflammatory arthritis globally, characterized by the deposition of monosodium urate (MSU) crystals in joints and periarticular tissues, leading to acute, painful flares of inflammation (1). The prevalence of gout has risen steadily worldwide, affecting an estimated 55.8 million people in 2020, with projections indicating an increase to 95.8 million by 2050 due to population aging and growth (2). The global burden of gout is substantial and increasing (2), with an estimated prevalence ranging from 1% to over 10% in certain populations (1), contributing significantly to disability and comorbid disease. Management of gout focuses on long-term urate-lowering therapy (ULT) to maintain serum urate levels below the saturation point (<6 mg/dL or 360 μmol/L), as recommended by international guidelines such as those from the American College of Rheumatology (ACR) and the European League Against Rheumatism (EULAR) (3, 4). Acute flares are typically managed with anti-inflammatory agents, including non-steroidal anti-inflammatory drugs (NSAIDs), colchicine, or glucocorticoid (3, 4).

Despite these strategies, a significant proportion of patients develop refractory gout, a condition particularly challenging when complicated by glucocorticoid dependence. Long-term or undisclosed glucocorticoid use can induce adrenal suppression and create a state of functional dependence. In such cases, dose reduction, if not managed with a very gradual taper, can be challenging and may precipitate acute adrenal insufficiency or flare recurrence, thereby perpetuating a cycle of dependency. Patients with refractory gout frequently have comorbidities such as chronic kidney disease (CKD), hypertension, or diabetes, further limiting treatment options (5).

The central role of the pro-inflammatory cytokine interleukin-1β (IL-1β) in the pathogenesis of acute gout flares provides a strong rationale for targeted therapy (6). Biologics like canakinumab, an anti-IL-1β monoclonal antibody, have shown efficacy in reducing pain and preventing flares in patients with contraindications to standard therapies (7). Firsekibart (formerly known as genakumab) is a novel, fully human monoclonal antibody that selectively neutralizes IL-1β with high affinity (8). Phase 1 studies in healthy volunteers demonstrated its safety and tolerability, while Phase 2 and 3 trials confirmed its non-inferiority to glucocorticoids (e.g., compound betamethasone) in acute pain relief and superiority in preventing flare recurrence over 12–24 weeks (8–10).

However, real-world evidence on the use of IL-1 inhibitors to facilitate glucocorticoid withdrawal in complex cases of glucocorticoid-dependent refractory gout remains limited. This represents a significant knowledge gap in the management of such challenging patients. This case report addresses this gap by describing the successful use of firsekibart, a novel anti-IL-1β antibody, to enable complete glucocorticoid discontinuation in a patient with severe tophaceous gout and apparent glucocorticoid dependence. We highlight firsekibart as a promising steroid-sparing agent in challenging gout scenarios, aligning with treat-to-target principles to improve long-term outcomes.

## Case presentation

A 53-year-old male presented to the emergency department on March 9, 2025, with a 3-week history of progressive pain, swelling, and ulceration of a tophus over his left first metatarsophalangeal (MTP) joint, accompanied by intermittent fever (peak temperature 38.9 °C). The patient had a body mass index (BMI) of 27.9 kg/m². He reported a long-standing diet high in seafood and beer prior to diagnosis, but had abstained from alcohol for the past two years. He had no history of smoking. He reported a 23-year history of gout since 2002, characterized initially by 2–3 acute flares annually involving the left first MTP joint, managed with over-the-counter analgesics. He had never received regular, supervised ULT. Over the preceding four years, the frequency of flares increased dramatically to more than 10 episodes per year. During this period, he self-medicated with an oral analgesic pill of unknown composition, which provided rapid symptomatic relief. His past medical history included hypertension for three years, managed with irbesartan 150 mg daily. There was no previously known history of diabetes or chronic kidney disease.

On admission, vital signs were: temperature 37.8 °C, pulse 149 bpm, blood pressure 126/85 mmHg. Physical examination revealed multiple tophi on the right elbow. The left first MTP joint was erythematous, markedly swollen, tender, and had a skin ulcer with yellowish purulent discharge. Initial laboratory tests showed leukocytosis (white blood cell count 14.62×10^9^/L) with neutrophilia (90.4%), markedly elevated high-sensitivity C-reactive protein (hs-CRP, 203.8 mg/L), hyperuricemia (uric acid 674 μmol/L), impaired renal function (creatinine 123.7 μmol/L, estimated glomerular filtration rate (eGFR) 57 mL/min/1.73 m²), and hyperglycemia (fingerstick blood glucose 14 mmol/L). A clinical diagnosis of infected tophus with cellulitis was made. He underwent urgent surgical debridement of the left foot ulcer and tophus removal.

He was admitted to the endocrinology department for further management. Additional workup confirmed type 2 diabetes (hemoglobin A1c 7.1%). Blood culture subsequently grew methicillin-resistant Staphylococcus aureus (MRSA). Antibiotic therapy was tailored accordingly, starting with ceftriaxone, then switching to moxifloxacin, and finally to vancomycin based on culture sensitivities and persistent fever. However, vancomycin was discontinued on March 26 due to the development of acute kidney injury (AKI; creatinine rising to 211.7 μmol/L, eGFR 30 mL/min/1.73 m²) and elevated trough levels (43.63 μg/mL). Anti-inflammatory therapy with colchicine (0.5 mg three times daily) and the NSAID loxoprofen was initiated. However, both agents were soon limited by worsening renal function (eGFR 28 mL/min/1.73 m²), which heightens the risk of colchicine toxicity and NSAID-related nephrotoxicity, and were discontinued on March 28.

The patient reported long-term use of unspecified analgesic pills that provided rapid relief of gout flares, suggesting possible undisclosed glucocorticoid content. Upon cessation of these pills after admission, he developed symptoms including fatigue, poor appetite, and recurrent fever, which raised the suspicion of withdrawal phenomena. Methylprednisolone (20 mg intravenously daily, later switched to oral) was therefore initiated to control both active inflammation and possible steroid withdrawal symptoms, allowing for a controlled taper. After methylprednisolone initiation, his fever subsided, and general symptoms improved. He was discharged on April 2 on methylprednisolone 8 mg twice daily, insulin, and antihypertensive agents. Febuxostat 40 mg daily was started shortly after discharge on April 10 as ULT and later increased to 80 mg daily.

Biochemical testing for adrenal insufficiency (e.g., serum cortisol or adrenocorticotropic hormone stimulation) was not performed during the acute admission due to the patient’s unstable condition and the urgent need to control infection and inflammation. Therefore, a definitive diagnosis of adrenal suppression could not be established.

Following discharge, multiple attempts to reduce the oral methylprednisolone dose below 16 mg per day (equivalent to 8 mg twice daily) were associated with acute gout flares in new joints: left knee in May 2025, right elbow and hand joints in June (after excessive lychee consumption), and left hand in August 2025. Each flare required re-escalation of the methylprednisolone dose or addition of meloxicam, with only transient benefit. The steroid tapering strategy was implemented in consultation with the endocrinology department, following general principles for glucocorticoid withdrawal in suspected dependency, although biochemical confirmation of adrenal insufficiency was not obtained. During this period, serum urate levels decreased from 674 μmol/L to around 490 μmol/L on febuxostat 80 mg daily but remained above the treatment target.

Due to these refractory flares and apparent glucocorticoid dependence, he was re-admitted in August 2025. On August 18, a single subcutaneous dose of firsekibart 200 mg (a recombinant anti- IL-1β monoclonal antibody) was administered. Following this intervention, no further gout flares occurred. This allowed for a progressive and successful taper of methylprednisolone: from 16 mg daily in August to 8 mg daily in September, then to 6 mg daily, and finally complete discontinuation by October 16, 2025. During this steroid taper, febuxostat was switched to dotinurad (initially 1 mg, then increased to 2 mg daily) for ongoing urate control.

**Figure 1:**
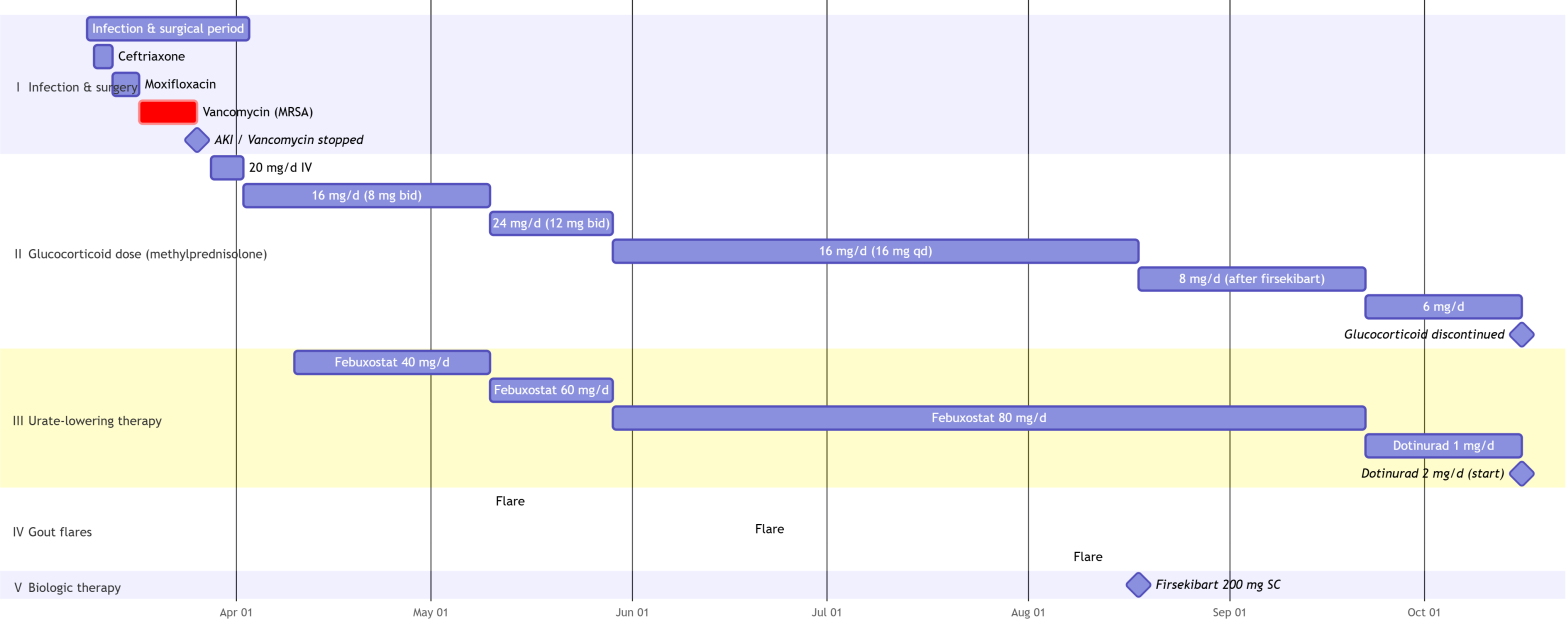
Clinical timeline of glucocorticoid−dependent refractory gout before and after firsekibart. AKI, acute kidney injury; bid, twice daily; IV, intravenous; MRSA, methicillin−resistant Staphylococcus aureus; qd, once daily; SC, subcutaneous.

After firsekibart administration, inflammatory markers normalized (hs-CRP decreased from 49.07 mg/L to 1.35-3.58 mg/L; erythrocyte sedimentation rate (ESR) decreased to 8.48 mm/h). Renal function recovered and stabilized (eGFR around 74–78 mL/min/1.73 m²). Serum urate levels progressively decreased to 425 μmol/L, although this remained above the recommended target of 360 μmol/L. The ulcer on the left first MTP healed completely with wound care. At the last follow-up in mid-October 2025, the patient was asymptomatic without glucocorticoids, on maintenance ULT (dotinurad 2 mg daily) and bone-protection therapy for osteoporosis diagnosed during admission. **Figure 1** provides a chronological overview of the clinical course, key interventions, and relevant laboratory trends from February to October 2025.

Throughout the management period, the patient received standardized lifestyle counselling, including dietary purine restriction, encouragement of low−fat dairy consumption, maintenance of adequate daily hydration (>2 L), and regular low−impact physical activity. He adhered to these recommendations, which may have contributed to flare prevention and overall metabolic control.

The longitudinal trends of key laboratory parameters throughout the clinical course—from initial admission to the final follow−up—are summarized in **Table 1**.

**Table 1:** Longitudinal changes of key laboratory parameters throughout the clinical course.

Parameter (reference range)	09 Mar (admission)	26 Mar (AKI peak)	02 Apr (Discharge)	13 May (1st flare)	15 Aug (pre-firsekibart)	22 Sep (1 month post)	16 Oct (2 months post)
hs-CRP (0–8.0 mg/L)	203.8	37.7	8.5	46.88	49.07	1.35	3.58
ESR (0–10 mm/h)	–	–	–	–	27.96	10.83	8.48
Serum urate (208–428 μmol/L)	674	698	631	510	490	453	425
Creatinine (58–110 μmol/L)	123.7	211.7	185.0	92.5	95.0	97.8	98.8
eGFR (≥80 mL/min/1.73m²)	57	30	35	81	78	76	74
WBC count (3.5–9.5×10^9^/L)	14.62	6.19	9.91	15.35	9.65	10.18	9.61

Reference ranges are derived from the hospital laboratory. eGFR was calculated using the CKD-EPI equation. AKI, acute kidney injury; hs-CRP, high-sensitivity C-reactive protein; ESR, erythrocyte sedimentation rate; eGFR, estimated glomerular filtration rate; WBC, white blood cell. “–” indicates that the test was not performed at that time point.

## Patient perspective

The patient provided the following statement regarding his experience: “I have had gout for over 20 years. In recent years, the attacks became so frequent that I could barely walk. I found some pills from a private pharmacy that stopped the pain within hours—I never knew they contained steroids. Every time my doctors tried to reduce the steroids, the pain came back. After I received the firsekibart injection in August, I have not had a single flare. I finally stopped steroids and can move freely. I am truly grateful.”

## Discussion

This case describes a patient with severe, treatment-refractory tophaceous gout whose clinical course was complicated by three intersecting challenges: life-threatening infection, intolerance to conventional anti-inflammatory therapies due to multiple comorbidities, and suspected glucocorticoid dependence (11). The successful discontinuation of glucocorticoids following a single dose of firsekibart highlights the potential of targeted IL-1β inhibition as a steroid-sparing strategy in this difficult-to-treat population.

Initial management appropriately prioritized control of an MRSA infection of an ulcerated tophus, requiring surgical debridement and targeted antibiotics. Once the infection was controlled, attention turned to the underlying inflammatory arthritis. However, standard pharmacologic options were severely constrained. Colchicine and NSAIDs were contraindicated due to AKI, hypertension, and newly diagnosed diabetes—conditions that increase the risk of nephrotoxicity and cardiovascular events with these agents (12). This therapeutic dead end necessitated reliance on glucocorticoids.

A critical diagnostic insight was the suspicion of surreptitious glucocorticoid use, based on the patient’s history of rapid relief from unidentified analgesic pills and the presence of classic steroid-induced comorbidities (osteoporosis, new-onset diabetes). Although biochemical confirmation of adrenal insufficiency was not possible during his unstable admission, the clinical picture—particularly the consistent recurrence of flares whenever methylprednisolone was tapered below 16 mg/day—strongly suggested a state of functional glucocorticoid dependence. This created a vicious cycle: steroid tapering precipitated flares, and flares necessitated continued steroid use, perpetuating both the inflammatory state and its metabolic consequences.

The central role of IL-1β in acute gouty inflammation provided a compelling rationale for targeted intervention. MSU crystals activate the NLRP3 inflammasome, leading to caspase-1-mediated cleavage of pro-IL-1β into its active form, which drives neutrophil recruitment, vasodilation, and pain (6, 13, 14). This pathway is now well-established as a therapeutic target, with several IL-1 inhibitors demonstrating efficacy in gout (7, 15, 16).

Firsekibart, a novel fully human monoclonal antibody that selectively neutralizes IL-1β with high affinity, has shown promise in recent trials (8). Phase II and III studies have demonstrated its non-inferiority to glucocorticoids (e.g., compound betamethasone) in resolving acute flares and its superiority in preventing flare recurrence over 12–24 weeks (9, 10, 17). In the pivotal GUARD-1 trial, a single dose of firsekibart reduced the risk of new flares by approximately 90% compared to steroid alone (9). These data position firsekibart as a highly effective option for both acute treatment and prophylaxis in patients unsuitable for standard therapies.

The definitive intervention, however, was the introduction of firsekibart. In our patient, the introduction of firsekibart appeared to break the cycle of glucocorticoid dependence. By potently and selectively blocking IL-1β, firsekibart likely suppressed the subclinical inflammation that was unmasked during steroid tapering. This created a “safe window” for gradual steroid withdrawal, as evidenced by the rapid and sustained normalization of hs-CRP and ESR following administration. The definitive intervention—firsekibart—enabled a careful, stepwise reduction of methylprednisolone over two months, culminating in complete discontinuation without a single flare. This outcome had been unattainable with conventional agents alone.

While flare suppression was achieved, optimal urate control remained elusive. The patient’s serum urate decreased from 674 μmol/L to 425 μmol/L on combination ULT (febuxostat followed by dotinurad) but never fell below the recommended treat−to−target threshold of 360 μmol/L (3, 4). This dissociation underscores a critical lesson: flare suppression and urate lowering are parallel, not sequential, therapeutic goals. The window created by IL-1β inhibition must now be leveraged to intensify ULT and achieve long−term disease modification.

The choice of ULT in this patient warrants comment. Febuxostat was selected over allopurinol due to the patient’s significant renal impairment at presentation (eGFR 57 mL/min/1.73 m²) and subsequent AKI, which would have necessitated complex allopurinol dose adjustments and raised concerns about hypersensitivity (18, 19). Despite dose escalation to 80 mg daily, febuxostat failed to achieve target urate levels. The subsequent switch to dotinurad, a selective urate transporter 1 inhibitor, was consistent with the 2025 Chinese Expert Consensus on Urate−Lowering Therapy for High−Risk Hyperuricemia, which recommends uricosurics when xanthine oxidase inhibitor monotherapy is insufficient, particularly in patients with CKD (20). Although dotinurad may offer renal protective benefits in some patients with CKD (21, 22), its independent contribution to flare suppression in this case cannot be isolated from the dominant effect of firsekibart; the immediate and complete cessation of flares after firsekibart—before dotinurad was introduced—strongly suggests that IL−1β inhibition was the primary driver. Losartan, which has mild urate−lowering effects (23), was not used in this patient as his blood pressure was well−controlled on irbesartan.

This report has several limitations. First, as a single case, causality cannot be definitively established, and findings are not generalizable. Second, glucocorticoid dependence was clinically suspected but not biochemically confirmed. Third, the switch from febuxostat to dotinurad introduces a confounding variable, though the temporal relationship favors firsekibart as the key intervention. Fourth, follow−up after complete steroid withdrawal is short (approximately two months), limiting conclusions about long−term remission and safety. Finally, serum urate remained above target (3, 4), indicating that optimal disease control was not fully achieved.

Despite these limitations, this case generates clinically relevant hypotheses. It highlights the need for a high index of suspicion for surreptitious glucocorticoid use in patients with refractory gout and a history of rapid, unexplained relief from unidentified medications. Persistent elevation of inflammatory markers (e.g., hs−CRP) during steroid tapering may identify patients at high risk for flare recurrence who could benefit from early IL−1 inhibition. Prospective randomized controlled trials are warranted to evaluate firsekibart as a steroid−sparing agent in this population, and long−term registries would provide real−world data on safety and durability of response.

In conclusion, this case provides proof−of−concept that a single dose of firsekibart can enable complete glucocorticoid withdrawal in a patient with severe, glucocorticoid−dependent refractory gout. By selectively blocking IL−1β, firsekibart suppressed the inflammatory drive that had previously prevented steroid tapering, creating a safe window for de−escalation. However, this case also serves as a reminder that flare control does not equate to disease control. The ultimate goal remains sustained urate lowering to below target (3, 4), and the window created by biologics like firsekibart should be used to intensify ULT and achieve long−term remission.

## Data Availability

The original contributions presented in the study are included in the article/supplementary material. Further inquiries can be directed to the corresponding authors.
